# Prevalence and predictors of resistant hypertension in a primary care setting: a cross-sectional study

**DOI:** 10.1186/1471-2296-15-131

**Published:** 2014-07-05

**Authors:** Yook Chin Chia, Siew Mooi Ching

**Affiliations:** 1 Department of Primary Care Medicine, University of Malaya Primary Care Research Group (UMPCRG), Faculty of Medicine, University of Malaya, 50603 Kuala Lumpur, Malaysia; 2 Curtin Health Innovation Research Institute, Faculty of Health Sciences, Curtin University, GPO Box U1987, 6845 Perth, Western Australia, Australia; 3 Department of Family Medicine, Faculty of Medicine and Health Sciences, Universiti Putra Malaysia, 43400 Serdang, Malaysia; 4 Department of Gerontology, Universiti Putra Malaysia, 43400 Serdang, Malaysia

**Keywords:** Resistant, Hypertension, Primary care, Prevalence, Predictors, Chronic kidney disease Malaysia

## Abstract

**Background:**

Patients with resistant hypertension are subjected to a higher risk of getting stroke, myocardial infarction, congestive heart failure and renal failure. However, the exact prevalence of resistant hypertension in treated hypertensive patients in Malaysia is not known. This paper examines the prevalence and determinants of resistant hypertension in a sample of hypertensive patients.

**Methods:**

We examined the control of blood pressure in a randomly selected sample of patients with hypertension in a primary care clinic. Demographic data, blood pressure and anti-hypertensive drug use were captured from patient records at the end of 2007. Resistant hypertension is defined as failure to achieve target blood pressure of < 140/90 mmHg while on full doses of an appropriate three-drug regimen that includes a diuretic. Multivariate logistic regression was used for the analysis.

**Results:**

A total of 1217 patients with hypertension were entered into the analysis. Mean age of the patients was 66.8 ± 9.7 years and 64.4% were female. More than half of the subjects (56.9%) had diabetes mellitus. Median BP was 130/80 mmHg. Overall prevalence of resistant hypertension was 8.8% (N = 107/1217). In multivariate logistic regression analysis, presence of chronic kidney disease is more likely to be associated with resistant hypertension (odds ratio [OR] 2.89, 95% confidence interval [CI] 1.56-5.35). On the other hand, increase per year of age is associated with lower odds of resistant hypertension in this population (OR 0.96, 95% CI 0.93-0.99).

**Conclusions:**

Resistant hypertension is present in nearly one in ten hypertensive patients on treatment. Hypertensive patients who have underlying chronic kidney disease are associated with higher odds of having resistant hypertension. Hence, in managing patients with hypertension, primary care physicians should be more alert and identify patients with chronic kidney disease as such patients are more likely to develop resistant hypertension. By doing that, these patients can be treated more aggressively earlier in order to achieve blood pressure target and thus reduce cardiovascular events.

## Background

Resistant hypertension is an important medical condition as uncontrolled blood pressure (BP) is associated with a fourfold risk of cardiovascular events compared with hypertensive patients achieving BP targets [1,2]. The definition of resistant hypertension varies. The Seventh Report of the Joint National Committee on Prevention, Detection, Evaluation, and Treatment of High BP defines resistant hypertension as failure to achieve BP to target despite adherence to appropriate treatment with full doses of at least 3 drug regimens including a diuretic [3]. On the other hand, the National Institute for Health and Care Excellence [4] and the European Society of Hypertension guidelines do not include the use of a diuretic in their definition [5].

Studies also show that resistant hypertension is associated with increasing age, female gender, black race, presence of diabetes, obesity, chronic kidney disease and left ventricular hypertrophy [6-14]. Early recognition of resistant hypertension followed by aggressive treatment is important to reduce both cardiovascular morbidity and mortality. However the exact prevalence of resistant hypertension is not precisely known due to its varied definition [3,15] and the setting of where the study was done. As such, based on several studies resistant hypertension has been reported to range from 5% to 50% [16-19]. Furthermore most studies on resistant hypertension were done in secondary care and very few have been conducted in primary care.

Strokes are more common in Asia than in developed countries while the reverse is true for coronary artery disease [20]. Elevated BP is one of the most important risk factors for stroke [21,22] and poorly controlled hypertension increases this risk [3,23,24]. Hypertension is of particular importance in South East Asia because while the prevalence of hypertension in Asian countries is nearly the same as that of most developed countries [25] , unfortunately many more patients in Asia with hypertension are not controlled to target [26,27] compared to developed countries. For example , in developed countries the control rates of hypertension was around 52% [28] to 60% [29] but it is as low as 26% in Malaysia [30]. Resistant hypertension is one of the contributors of uncontrolled hypertension. Little is known about the prevalence of resistant hypertension in the South East Asian population. Hence we wanted to examine prevalence of resistant hypertension in treated hypertensive patients in a primary care setting in a South East Asian country to ascertain its contribution to uncontrolled hypertension.

## Methods

### Setting

We examined the control of blood pressure in a randomly selected sample of patients in an urban primary care clinic. Random numbers was generated by computer based on the patient registration number with the clinic. This selected sample consists of adult patients aged 30 years and older with hypertension who were treated and on long term follow-up in our primary care clinic. The study was conducted in an outpatient clinic of the University Malaya Medical Centre, a teaching hospital in Kuala Lumpur, the capital city of Malaysia. This clinic is run by 14 family medicine specialists, 30 vocational trainees in family medicine and other medical officers. This tertiary hospital including its primary care clinic serves a multi-ethnic population of 450,000 in the surrounding area.

People in the community can choose their own source of care. This teaching hospital functions on an open access basis to the community. A full range of services are offered at this clinic. Those who attend our clinic are mainly those requiring long term care of chronic conditions like hypertension, diabetes, dyslipidaemia in addition to the usual short term primary care illnesses.

Care is provided free for public sector service employees and their dependents while all others are required to pay an equivalent of US$8.50 for both the consultation and a month’s supply of medication. Our study population consists of three main ethnic groups, namely the Malays, Chinese and Indians [31].

Ethics approval for this study was obtained from the Ethics Committee of the University of Malaya Medical Centre.

### Inclusion criteria

All patients with underlying hypertension were eligible for this study. Hypertension was defined as those who had a documented diagnosis of hypertension [i.e. BP ≥ 140/90 mmHg] or those on anti-hypertensive agents.

### Data collection

This sample was randomly selected using a computer generated number based on the patient’s unique registration number with the clinic. All patient records were in paper form. We extracted the patients’ information based on the last entry in 2007 from their records manually according to a predetermined proforma (clinical report form) which included the patient’s socio demographic, blood pressure, weight, height, biochemical profile and use of anti-hypertensive agents. This was then entered into an electronic Excel spreadsheet and then converted to a SPSS format for analysis using SPSS version 21.

The data was captured by a trained and experienced abstractor and accuracy of data entry was checked by the investigators themselves.

Patients’ blood pressure which was measured by the attending doctors as part of daily routine care were also captured from the medical records. Patients recruited into our study were those who had been diagnosed to have hypertension or were on antihypertensive medications. Diagnosis of hypertension in our clinics is made in accordance with standard recommendations i.e. based on at least 2 BP measurements at least 2 weeks apart [3]. Height and weight had been recorded using a stadiometer and digital weighing machine. We computed the body mass index (BMI) as weight in kilograms per square meter height (kg/m2).

We used the Cockcroft-Gault formula to calculate the estimated glomerular filtration rate (GFR) as a measure of renal function. The presence of chronic kidney disease (CKD) was defined as estimated GFR < 60 mL/min per 1.73 m^2^.

Anti-hypertensive drug use was also captured from medical records and classified into the following classes: renin-angiotensin-system (RAS) inhibitors encompassing angiotensin-converting enzyme inhibitors (ACEI), angiotensin receptor blockers (ARB); beta-blockers, calcium-channel blocker (CCB), diuretics and alpha-blockers N.

Resistant hypertension in this study is defined as office BP ≥140 and/or 90 mmHg despite the use of at least three antihypertensive drugs, one of which is a diuretic. Diabetes mellitus (DM) was based on the doctors’ diagnosis or the use of hypoglycemic agents or both as stated in the medical records. Smokers were defined as current if they were still smoking; non smokers for those who never smoked or currently not smoking, regardless of when they had stopped smoking as indicated in the patient records.

### Statistical analysis

All statistical analysis was done using the Statistical Package for Social Sciences (SPSS version 21). Continuous data are described as mean and standard deviation if the distribution is normal. When the data was a skewed distribution, median, minimum and maximum value were used to describe the data. Categorical data are reported as proportions (percentage). Chi-square test or Fisher exact tests were used for the categorical or dichotomous variables. Multivariate logistic regression analysis was used to look for the predictors of resistant hypertension. All variables with the p-value of less than 0.25 in the univariate analyses as well as clinically significant variables were entered into the multivariate logistic regression. The dependent variable was resistant hypertension (yes or no). The independent variables were age, gender, ethnicity, BMI, presence of diabetes, chronic kidney disease, dyslipidaemia and smoking status. All analyses were done with 95% confidence intervals (CI), and the level of significance was set at p < 0.05.

## Results

A total of 1222 hypertensive patients were eligible for this study. Out of this, 1217 (99.6%) patients had complete drug therapy data and were entered into the analysis.

Table 1 shows the clinical characteristics of the studied population. Overall, the mean age of the patients was 66.8 ± 9.7 years; about two-thirds were female (66.4%), and 59% were aged more than 65 years. The median BP was 130/80 mmHg. More than half of the populations (56.9%) were diabetics with a mean HbA1c of 7.0 ± 1.7%. The mean eGFR of the studied population was 62.7 ± 33.2 mL/ min per 1.73 m^2^ and a third (34.9%) of the patients had underlying CKD.

**Table 1:** Clinical characteristics of the studied population in UMMC (N = 1217)

**Characteristic of the subjects**	
Mean Age ± SD ,years	66.8 ± 9.7
Female, n, %	784 (64.4)
Ethnicity, n, %	
Malays	312 (25.6)
Chinese	540 (44.4)
Indians	348 (28.6)
Systolic blood pressure (IQR), mmHg	130 (90–220)
Diastolic blood pressure (IQR), mmHg	80 (50–110)
Resistant hypertension, n, %	107 (8.8)
Presence of DM, n, %	693 (56.9)
Missing value, n %	20 (1.6%)
BMI (IQR), kg/m2	25.8 (12.9-55.5)
Missing value, n, %	367 (30.2)
presence of CKD, n, %	425 (34.9)
Missing value, n, %	261 (21.4)
Smoker, n, %	86 (7.1)

IQR: interquartile range.

The mean number of anti-hypertensive agents used was 2.0 ± 1.0. The control rate of those on 2 drugs was 52.3% while it was 48.0% for those on 3 drugs. Three fifths (60.9%) of the diabetics and two fifths (41.3%) of the patients with CKD were on 3 antihypertensive agents. Calcium-channel blockers were the most commonly prescribed drug (53.2%) followed by angiotensin converting enzyme inhibitors or angiotensin receptor blockers (51.0%), beta-blockers (44.5%), diuretics (32.5%) and alpha-blockers (3.5%). For those patients who were on at least three drugs, 70.2% (n = 181) were on diuretics and 29.8% (n = 77) were not on diuretics. Among those patients who were on at least 3-antihypertensive agents including diuretics, 40.9% (n = 74) achieved blood pressure target while 59.1% (n = 107) had resistant hypertension. On the other hand, of those on at least 3 antihypertensive drugs which did not include a diuretic 96.1%, (n = 74) were controlled and 3.9% (n = 3) were not controlled.

Overall prevalence of resistant hypertension was 8.8% (N = 107) with 95% confidence interval (CI) 7.21-10.39. Table 2 compares the characteristics of those with and without resistant hypertension.

**Table 2:** Association between clinical variables among patients with and without resistant hypertension

**Variables**	**No resistant HPT (91.2%) (n = 1110)**	**Resistant HPT (8.8%) (n = 107)**	**p**
Age (years)	66.9 ± 9.5	65.5 ± 9.5	0.15
Female, n, %	713 (64.2)	71 (66.4)	0.66
Ethnicity, n , %			
Malay	281 (25.4)	31 (29.0)	0.99
Chinese	495 (44.7)	45 (42.1)	
Indians	319 (28.8)	29 (27.1)	
BMI, kg/m2	26.3 ± 4.8	27.4 ± 5.4	0.08
Diabetes mellitus, n, %	672 (61.3)	67 (62.6)	0.79
Dyslipidaemia, n, %	734 (66.1)	76 (71.0)	0.34
Chronic kidney disease, n, %	377 (43.5)	48 (53.3)	0.08
Smoker, n, %	77 (6.9)	9 (8.4)	0.93

HPT: hypertension, n: number, BMI: body mass index.

Table 3 shows the results of multivariate logistic regressions. After adjusting for all the variables in the model, age and presence of CKD were the main predictors. Presence of CKD among patients with hypertension were more likely to be associated with resistant hypertension compared to those without CKD (odds ratio [OR] 2.89, 95% CI 1.56-5.35). Increasing age is less likely to be associated with resistant hypertension (OR 0.96, 95% CI 0.93-0.99).

**Table 3:** Predictors of resistant hypertension at UMMC (N = 1217)

**Variables**	Adjusted OR*	**95% C.I.**		**p value**
		**Lower**	**Upper**	
Chronic kidney disease	**2.89**	1.56	5.35	0.001**
Body mass index (per 1 kg/m2 increase)	**1.05**	0.99	1.10	0.08
Age (per 1 year increase)	**0.96**	0.93	0.99	0.007**
Diabetes	**0.88**	0.51	1.51	0.64
Ethnicity				
Malays	**1.30**	0.64	2.63	0.47
Chinese	**1.20**	0.62	2.34	0.59
Indians	**1.00**			
Smoking	**1.01**	0.20	5.07	0.99
Dyslipidaemia	**1.33**	0.73	2.42	0.36
Gender				
Female	**1.57**	0.90	2.74	0.11
Male	**1.00**			

*Adjusted odds ratio CI: Confidence Interval. **p-value is <0.05.

## Discussion

The prevalence of resistant hypertension in this primary care setting is 8.8%. This is lower than that reported in secondary care [8,32,33] but is similar to another primary care study [18].

Our findings also show that hypertensive patients with CKD had 2.9 odds of having resistant hypertension compared to those without CKD. This is consistent with findings in other studies [12,14]. One possible reason is that in patients with CKD, there is increased sensitivity to salt resulting in sodium and fluid retention, thus making BP more difficult to control [34]. Another possible explanation is that in CKD, the RAS system is up regulated resulting in difficult to control BP [35].

Our study sample is made up of older patients as seen from the mean age of 66.9 years. That resistant hypertension is negatively associated with age could be due to the survival effect of fitter patients, whereby those with resistant hypertension had already succumbed to the complications of uncontrolled or resistant hypertension.

Previous studies have shown obesity to be associated with resistant hypertension [3], although we only found weak evidence (p = 0.08) for such an association. A possible reason could be because of our small sample size of patients with resistant hypertension as the difference in body mass index between patient with and without hypertension resistant is not great (27.4 kg/m^2^ versus 26.3 kg/m^2^).

Furthermore studies have shown diabetes to be associated with resistant hypertension [1,8,12] but we could not find any association in our study. This could be due to only a small difference in HbA1c between these two groups, where the mean HbA1c was 7.9 ± 2.0% among patients with resistant hypertension versus HbA1c of 7.5 ± 1.7% in patients without resistant hypertension, p = 0.139).

Many studies have shown that most patients with hypertension need 2 or more drugs to achieve target BP [36-38]. Not surprisingly because the mean number of antihypertensive drugs used in our study was 2. We also found poor control rates amongst those on only 2 drugs, even those on 3 drugs had lower than 50% control rate. When control is not to target particularly when patients are already on 3 drugs, the recommendation is that one of the drugs should be a diuretic. However we found that overall the use of diuretics was low. The definition of resistant includes use of a diuretic. Therefore, those patients on triple antihypertensive therapy but not including a diuretic, are not considered to have resistant hypertension in this study. This may therefore result in an underestimation of the true prevalence of resistant hypertension in our population.

In our study, it is possible that interaction between some of the factors of interest may explain the stronger associations evident in the multivariate compared with univariate regressions, particularly for CKD. However, this was not evident when interaction terms were included between CKD and BMI (p = 0.79) but significant interaction found between CKD and age (p < 0.001).

### Strength and limitations

Our present study has several strengths and some limitations. The strength of our study is that it is done in the primary care setting where the prevalence of resistant hypertension is different from that in secondary care. Secondly, our sample size is large enough to give us a better picture of the prevalence of resistant hypertension in primary care.

A limitation of our study is that adherence to medication was not available from the patient records. If adherence is taken into account, the “true” resistant hypertension may be lower as many studies have shown that “resistant hypertension” is frequently due to non-adherence. However doctors in routine daily clinical practice are not accurate in assessing adherence and even if adherence is assessed, this information is seldom recorded. Pill counting or use of electronic devices is indicated to confirm adherence, but this may not be practical in a real clinical setting [39-41].

## Conclusions

The prevalence of resistant hypertension in this primary care population of patients with hypertension is relatively low. Nevertheless every effort is still needed to recognize it early. The presence of CKD is associated with higher odds of having resistant hypertension. On the other hand, increase in one year of age is associated with lower odds of having resistant hypertension in this population. These results indicate that clinicians should recognize resistant hypertension earlier, especially in those who have CKD so that early referral or intensifying therapy can be put in place.

## Abbreviations

BP: Blood pressure; DM: Diabetes mellitus; BMI: Body mass index; Kg/m2: Kilograms per square meter; eGFR: Estimated glomerular filtration rate; N: Number; CKD: Chronic kidney disease; CI: Confidence intervals; OR: Odds ratio; RAS: Renin-angiotensin-system.

## Competing interests

The authors declare that they have no competing interests.

## Authors’ contributions

CYC contributed in the conceptualization of the paper, data entry and writing of the manuscript while CSM contributed in data analysis and writing of the manuscript. CYC is the corresponding author. Both the authors read and approved the final manuscript.

## Pre-publication history

The pre-publication history for this paper can be accessed here:

http://www.biomedcentral.com/1471-2296/15/131/prepub
